# Update: Influenza Activity — United States, September 29, 2013–February 8, 2014

**Published:** 2014-02-21

**Authors:** Carmen S. Arriola, Lynnette Brammer, Scott Epperson, Lenee Blanton, Krista Kniss, Desiree Mustaquim, Craig Steffens, Rosaline Dhara, Michelle Leon, Alejandro Perez, Sandra S. Chaves, Jackie Katz, Teresa Wallis, Julie Villanueva, Xiyan Xu, Anwar Isa Abd Elal, Larisa Gubareva, Nancy Cox, Lyn Finelli, Joseph Bresee, Michael Jhung

**Affiliations:** 1 EIS officer, CDC; 2 Influenza Division, National Center for Immunization and Respiratory Diseases, CDC

Influenza activity in the United States began to increase in mid-November and remained elevated through February 8, 2014. During that time, influenza A (H1N1)pdm09 (pH1N1) viruses predominated overall, while few B and A (H3N2) viruses were detected. This report summarizes U.S. influenza activity* during September 29, 2013–February 8, 2014, and updates the previous summary (1).†

## Viral Surveillance

**FIGURE 1:**
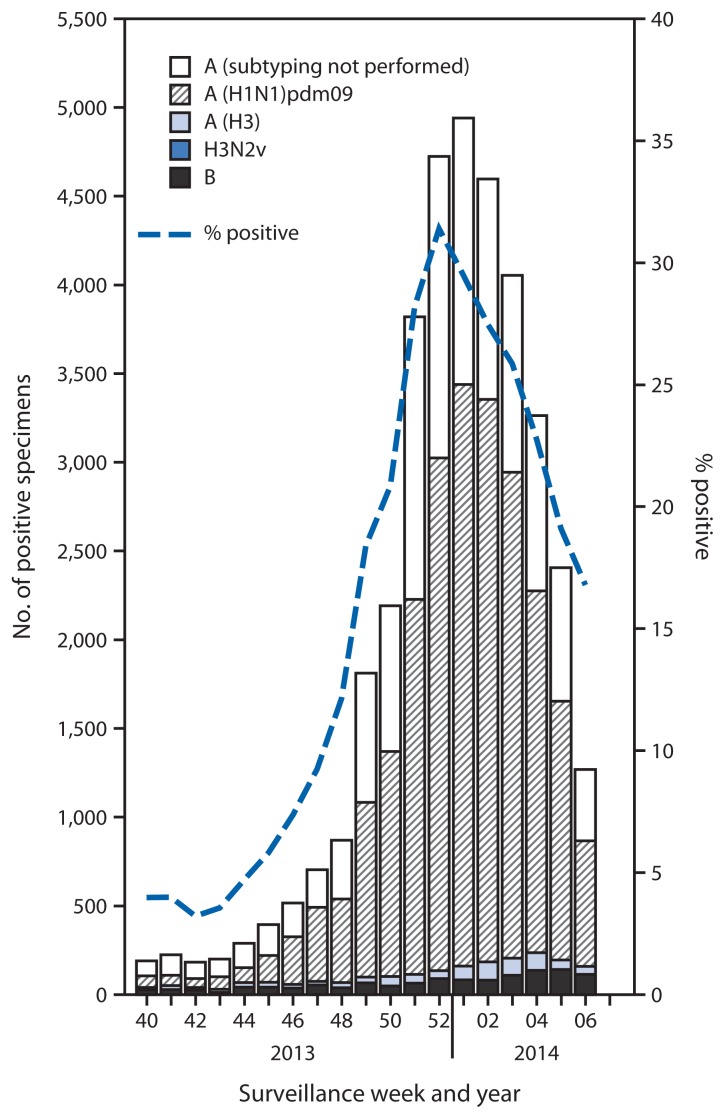
Number* and percentage of respiratory specimens testing positive for influenza, by type, surveillance week, and year — U.S. World Health Organization and National Respiratory and Enteric Virus Surveillance System collaborating laboratories, United States, 2013–14 influenza season^†^ * N = 36,619. ^†^ Data reported as of February 14, 2014.

During September 29, 2013–February 8, 2014, approximately 140 World Health Organization (WHO) and National Respiratory and Enteric Virus Surveillance System collaborating laboratories in the United States tested 189,123 respiratory specimens for influenza viruses; 36,619 (19%) were positive (Figure 1). Of these, 35,365 (97%) were influenza A viruses, and 1,254 (3.4%) were influenza B viruses. Of the 35,365 influenza A viruses, 23,111 (65%) were subtyped; 845 (3.7%) of these were influenza A (H3) viruses, one was H3N2v, and 22,265 (96%) were pH1N1. Since September 29, 2013, influenza-positive tests have been reported from all 50 states, the District of Columbia, and Puerto Rico, representing all 10 U.S. Department of Health and Human Services regions.§ The percentage of specimens testing positive for influenza increased through the week ending December 28, 2013 (week 52), when 31% tested positive, and decreased subsequently. In the week ending February 8, 2014 (week 6), 17% of specimens tested positive. Since the start of the influenza season, through February 8, 2014, pH1N1 viruses predominated in the United States overall; influenza A (H3N2) and B viruses were identified infrequently.

## Antigenic Characterization

WHO collaborating laboratories in the United States are requested to submit a subset of their influenza-positive respiratory specimens to CDC for further antigenic characterization. CDC has antigenically characterized 1,046 influenza viruses collected by U.S. laboratories during the 2013–14 season, including 920 pH1N1 viruses, 86 influenza A (H3N2) viruses, and 40 influenza B viruses. Of the 920 pH1N1 viruses, 919 were characterized as A/California/7/2009-like, which is the influenza A (H1N1) component of the 2013–14 Northern Hemisphere vaccine. All influenza A (H3N2) viruses were antigenically like A/Texas/50/2012, which is the influenza A (H3N2) component of the 2013–14 Northern Hemisphere vaccine. Of the 40 influenza B viruses tested, 21 (52.5%) belong to the B/Yamagata lineage and were characterized as B/Massachusetts/2/2012-like, which is included as an influenza B component in the 2013–14 Northern Hemisphere trivalent and quadrivalent influenza vaccines. The remaining 19 (47.5%) influenza B viruses tested belong to the B/Victoria lineage and were characterized as B/Brisbane/60/2008-like, which is included as an influenza B component in the 2013–14 Northern Hemisphere quadrivalent influenza vaccine.

## Antiviral Resistance of Influenza Viruses

Testing of pH1N1, influenza A (H3N2), and influenza B virus isolates for resistance to neuraminidase inhibitors (oseltamivir and zanamivir) is performed at CDC and public health laboratories using a functional assay. Additional pH1N1 and influenza A (H3N2) clinical samples are tested for mutations of the virus known to confer oseltamivir resistance. Since October 1, 2013, a total of 3,314 influenza viruses have been tested for antiviral resistance, including 3,109 pH1N1 viruses, 151 influenza A (H3N2) viruses, and 54 influenza B viruses. Of the 3,109 pH1N1viruses tested, 25 (0.8%) were resistant to oseltamivir. Of the 1,120 pH1N1 viruses tested for resistance to zanamivir, all (including all oseltamivir-resistant viruses tested) were sensitive to zanamivir. All influenza A (H3N2) and influenza B viruses tested were sensitive to both oseltamivir and zanamivir.

## Outpatient Illness Surveillance

**FIGURE 2:**
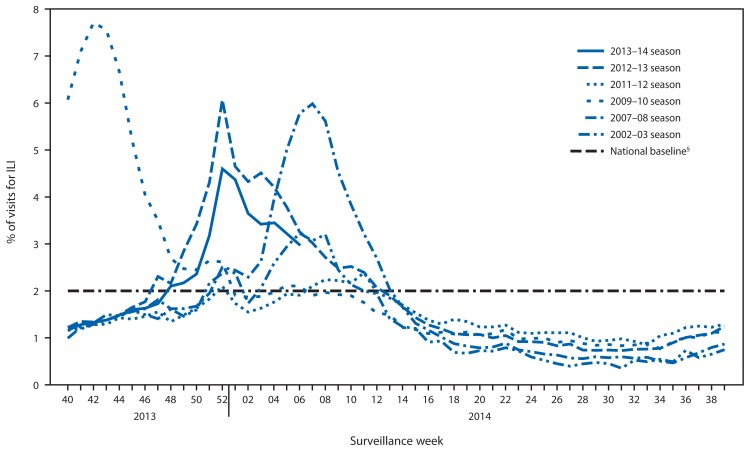
Percentage of all outpatient visits that are for influenza-like illness (ILI)* reported to CDC, by surveillance week — Outpatient Influenza-Like Illness Surveillance Network, United States, September 29, 2013–February 8, 2014, and selected previous influenza seasons^†^ * Defined as a fever (≥100°F [≥37.8°C]), oral or equivalent, and cough and/or sore throat, without a known cause other than influenza. ^†^ Data reported as of February 14, 2014. ^§^ The national baseline is the mean percentage of visits for ILI during weeks with little or no influenza virus circulation (noninfluenza weeks) for the previous three seasons plus two standard deviations. A noninfluenza week is defined as periods of ≥2 consecutive weeks in which each week accounted for <2% of the season’s total number of specimens that tested positive for influenza. National and regional percentages of patient visits for ILI are weighted on the basis of state population. Use of the national baseline for regional data is not appropriate.

Since September 29, 2013, the weekly percentage of outpatient visits for influenza-like illness (ILI)¶ reported by approximately 2,000 U.S. Outpatient ILI Surveillance Network (ILINet) providers in all 50 states, New York City, Chicago, the U.S. Virgin Islands, Puerto Rico, and the District of Columbia, which comprise ILINet, has ranged from 1.2% to 4.6% and was at or above the national baseline** of 2.0% from the week ending November 30, 2013 (week 48) to February 8, 2014 (week 6) (Figure 2). Peak weekly percentages of outpatient visits for ILI ranged from 2.4% to 7.6% from the 1997–98 through 2012–13 seasons, excluding the 2009 pandemic. For the week ending February 8, 2014 (week 6), all 10 regions reported ILI activity above their region-specific baseline levels. This is the 14th week this season during which one or more region-specific baselines were exceeded. Data collected in ILINet are used to produce a measure of ILI activity†† by jurisdiction. During week 6, six states (Arkansas, Connecticut, Kansas, New York, Oklahoma, and Texas) experienced high ILI activity, seven states experienced moderate ILI activity (Alabama, Delaware, Hawaii, Louisiana, Maryland, New Jersey, and Virginia), and 19 states and New York City (Arizona, California, Colorado, Florida, Kentucky, Massachusetts, Minnesota, Mississippi, Missouri, Nebraska, New Mexico, Nevada, North Carolina, Pennsylvania, Rhode Island, South Dakota, Utah, Washington, and Wisconsin) experienced low ILI activity. ILI activity was minimal in 18 states, and data were insufficient to calculate an ILI activity level for the District of Columbia.

## Geographic Spread of Influenza Activity

For the week ending February 8, 2014 (week 6), the geographic spread of influenza§§ was reported as widespread in 24 states (Arizona, Arkansas, California, Connecticut, Delaware, Florida, Illinois, Indiana, Maine, Maryland, Massachusetts, Missouri, New Hampshire, New Jersey, New York, North Carolina, Ohio, Oklahoma, Pennsylvania, Rhode Island, South Carolina, Vermont, Virginia, and Utah), regional in 20 states (Alabama, Alaska, Colorado, Idaho, Iowa, Kansas, Kentucky, Louisiana, Montana, Michigan, Minnesota, Nebraska, Nevada, North Dakota, Oregon, South Dakota, Tennessee, Texas, Washington, and Wisconsin), and local in five states (Georgia, Mississippi, New Mexico, West Virginia, and Wyoming), Guam, and the District of Columbia. Sporadic influenza activity was reported by one state (Hawaii) and Puerto Rico. No influenza activity was reported by the U.S. Virgin Islands.

## Influenza-Associated Hospitalizations

**FIGURE 3:**
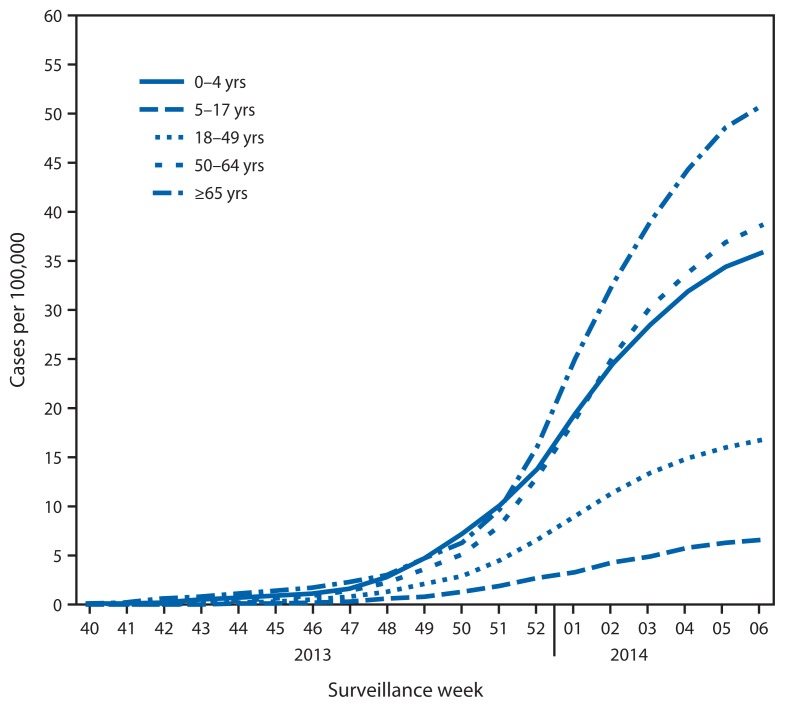
Rates of hospitalization for laboratory-confirmed influenza, by age group and surveillance week — FluSurv-NET,* 2013–14^†^ * FluSurv-NET conducts population-based surveillance for laboratory-confirmed influenza-associated hospitalizations in children aged <18 years (since the 2003–04 influenza season) and adults aged ≥18 years (since the 2005–06 influenza season). The FluSurv-NET covers approximately 80 counties in the 10 Emerging Infections Program states (California, Colorado, Connecticut, Georgia, Maryland, Minnesota, New Mexico, New York, Oregon, and Tennessee) and additional Influenza Hospitalization Surveillance Project states (Michigan, Ohio, and Utah). ^†^ Data reported as of February 14, 2014.

**FIGURE 4:**
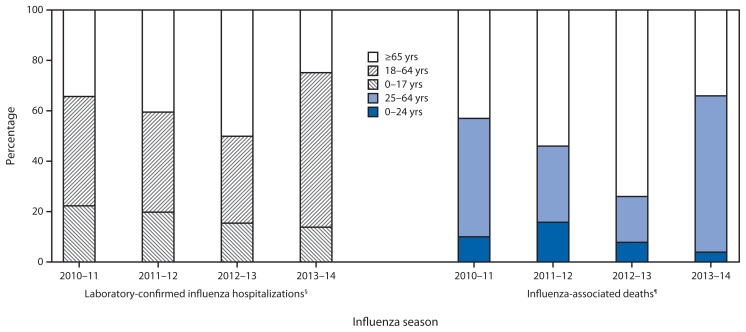
Percentage of laboratory-confirmed influenza hospitalizations and influenza-associated deaths, by age group* and influenza seasons^†^ — FluSurv-NET and 122 U.S. Cities Mortality Reporting System, United States, 2010–14 influenza seasons * For laboratory-confirmed influenza hospitalizations, age groups are 0–17 years, 18–64 years, and ≥65 years; for influenza-associated deaths, age groups are 0–24 years, 25–64 years, and ≥65 years. ^†^ Influenza A (H3N2) was the predominant virus circulating during the three previous seasons, while influenza A (H1N1)pdm09 has been the predominant virus circulating during the 2013–14 influenza season. For the 2013–14 season, all values presented are early estimates or data through week 6 of the influenza season; for previous seasons, data shown are end-of-season values. ^§^ Totals were 6,307 for the 2010–11 season, 2,409 for the 2011–12 season, 12,371 for the 2012–13 season, and 6,655 through February 8, 2014, for the 2013–14 season. ^¶^ Totals were 406 for the 2010–11 season, 122 for the 2011–12 season, 753 for the 2012–13 season, and 571 through February 8, 2014, for the 2013–14 season.

CDC monitors hospitalizations associated with laboratory-confirmed influenza in adults and children through the Influenza Hospitalization Surveillance Network (FluSurv-Net),¶¶ which covers approximately 27 million persons, 8.5% of the U.S. population. From October 1, 2013 through February 8, 2014 (week 6), a total of 6,655 laboratory-confirmed influenza-associated hospitalizations were reported. This yields a rate of 24.6 hospitalizations per 100,000 population (Figure 3). Persons aged ≥65 years had the highest influenza-associated hospitalization rate (50.9 per 100,000), followed by those aged 50–64 years (38.7 per 100,000), 0–4 years (35.9 per 100,000), 18–49 years (16.8 per 100,000), and 5–17 years (6.6 per 100,000). Of the 6,655 influenza-associated hospitalizations that have been reported, 9.4% were reported in persons aged 0–4 years, 4.5% in those aged 5–17 years, 61.2% in those aged 18–64 years, and 24.8% in those aged ≥65 years (Figure 4). Among cases, 6,328 (95%) were associated with influenza A virus infection, 253 (3.8%) were associated with influenza B, 21 (0.3%) were associated with influenza A and B coinfections, and 53 (0.8%) had no virus type information. Among those with influenza A subtype information, 39 (1.4%) were associated with influenza A (H3), and 2,766 (98.6%) were pH1N1.

The frequency distribution of chronic underlying medical conditions among hospitalized patients is based on a subset (approximately 30%) of cases with complete medical chart abstraction and may change as new data become available. Among hospitalized adults, 15% had no identified chronic underlying medical conditions, compared with 43% percent of hospitalized children. The most commonly reported chronic underlying medical conditions in adults were obesity (43%), metabolic disorders (33%), cardiovascular disease (29%), and chronic lung disease (excluding asthma) (27%). In children (persons aged <18 years), the most commonly reported chronic underlying medical conditions were asthma (24%), neurologic disorders (13%), obesity (10%), and chronic lung disease (excluding asthma) (8%). Among 301 hospitalized women of childbearing age (15–44 years), 65 (22%) were pregnant.

## Pneumonia and Influenza-Associated Mortality

During the week ending February 8, 2014 (week 6), pneumonia and influenza (P&I) was reported as an underlying or contributing cause for 8.4% (1,023 of 12,180) of all deaths reported to the 122 Cities Mortality Reporting System. This percentage is above the epidemic threshold*** of 7.3% for that week. Since September 29, 2013, the weekly percentage of deaths attributed to P&I ranged from 5.3% to 8.7%. The percentage first exceeded the epidemic threshold during the week ending January 11, 2014 (week 2) and remained elevated through the week ending February 8, 2014 (week 6). Peak weekly percentages of deaths attributable to P&I in the previous five seasons ranged from 7.9% during the 2008–09 and 2011–12 seasons to 9.9% during the 2012–13 season.

Among 14,628 P&I deaths reported through the 122 Cities Mortality Reporting System from September 29, 2013 to February 8, 2014, a total of 571 (3.9%) were influenza-associated (i.e., they had influenza listed on the death certificate as an underlying or contributing cause of death), of which 352 (62%) were in persons aged 25–64 years, 194 (34%) in persons aged ≥65 years, and 25 (4%) in persons aged 0–24 years (Figure 4).

## Influenza-Associated Pediatric Mortality

As of February 8, 2014 (week 6), 50 influenza-associated pediatric deaths that occurred in the 2013–14 season were reported to CDC: one was associated with an influenza B virus, 29 with pH1N1 viruses, 17 with an influenza A virus for which no subtyping was performed, one with an influenza A and influenza B virus coinfection, and two with an influenza virus for which the type was not determined. Since influenza-associated pediatric mortality became a nationally notifiable condition in 2004, the total number of influenza-associated pediatric deaths has ranged from 35 to 171 per season, excluding the 2009 pandemic, when 348 pediatric deaths were reported to CDC during April 15, 2009–October 2, 2010.

### Editorial Note

Influenza activity in the United States began to increase in mid-November and remained elevated and widespread as of February 8, 2014. During September 29, 2013–February 8, 2014, pH1N1 accounted for the majority of circulating influenza viruses, but influenza A (H3N2) and influenza B viruses also were identified. This season, influenza activity first increased in the southern states. By the end of December 2013, high influenza activity was seen throughout the United States. During the first 4 weeks of 2014, influenza activity decreased in the southeast and south central areas of the United States but began increasing in the west and northeast areas. Elevated influenza activity in parts of the United States is expected for several more weeks.

Surveillance data from previous influenza seasons have shown that the epidemiology of influenza is related to the circulating subtype, which can vary by season. This is the first season that pH1N1 has been the predominant influenza virus circulating in the United States since this subtype emerged in 2009. Although illness was seen in all age groups during the 2009 pandemic, persons aged 50–64 years had the highest influenza-associated death rate and second highest influenza-associated hospitalization rate among all age groups (2). Preliminary surveillance data for the 2013–14 influenza season suggest that although overall disease prevalence is lower than during the 2009 pandemic, persons aged 18–64 years are again at relatively high risk for severe illness from influenza this season. As of February 8, 2014, persons aged 18–64 years represented 4,077 (61%) of influenza-associated hospitalizations reported by FluSurv-NET. In contrast, during the past three seasons in which H3N2 or B influenza viruses predominated, persons aged 18–64 years accounted for only 35% (2012–13), 40% (2011–12), and 43% (2010–11) of all influenza-associated hospitalizations reported by FluSurv-NET (Figure 4). For the 2013–14 season, cumulative influenza-associated hospitalization rates for persons aged 18–49 years (16.8 per 100,000) and 50–64 years (38.7 per 100,000) in FluSurv-NET have already surpassed the end-of-season rates from three of the previous four seasons (3).

During the three previous influenza seasons, the total number of P&I deaths reported through the 122 Cities Mortality Reporting System ranged from 37,444 to 41,708, of which <1% to 2% were deaths for which influenza was listed on the death certificate as an underlying or contributing cause of death. Although the age distribution of pneumonia deaths this season is similar to previous seasons, the age distribution of influenza deaths has changed. The number of influenza deaths during the current season (through February 8, 2014) among persons aged 25–64 years (352) exceeds the 138 deaths reported for that age group for the entire 2012–13 influenza surveillance season (September 30, 2012–September 28, 2013). This age group has accounted for approximately 62% of all influenza-associated deaths already this season, compared with 47% in 2010–11, 30% in 2011–12, and 18% in 2012–13 (Figure 4).

The more severe impact of pH1N1 on adults aged 18–64 years seen this season and during the pandemic is thought to result from at least two factors. First, persons in this age group likely lack the cross-protective immunity to pH1N1 seen in adults aged ≥65 years, which was likely acquired from past infection with antigenically related viruses (4). Second, preliminary vaccination coverage estimates for this season indicate that by early November 2013, adults aged 18–64 years had been vaccinated against influenza at a rate substantially lower (33.9%; 95% confidence interval [CI] = 31.9%–35.9%) than those aged 6 months–17 years (41.1%; 95% CI = 38.8%–43.4%) and ≥65 years (61.8%; 95% CI = 57.9%–65.7%) (5). In previous years, adults aged 18–64 years also have been less likely to receive influenza vaccine, compared with persons in other age groups (5). Although some persons infected with pH1N1 during the 2009 pandemic might retain some residual immunity, this protection has likely declined over time. Furthermore, seroprevalence studies showed that only a minority (approximately 35% of all ages combined) were seropositive for pH1N1 after the 2009 pandemic, with even smaller percentages (26%) among those aged 25–64 years (6).

Surveillance data available from the 2013–14 season are a reminder that, although some age groups are at increased risk of influenza complications every year (e.g., adults aged ≥65 years), influenza can cause severe illness in persons of any age, even in adults aged 18–64 years. Vaccination is the primary means to prevent influenza and its complications and is recommended annually for all persons aged ≥6 months. Data from the current and two previous influenza seasons suggest that vaccination reduced the risk for medical visits associated with influenza by 47%–61% (7,8). Health-care providers should continue to recommend and offer influenza vaccine for the remainder of the season to all unvaccinated persons aged ≥6 months.

Early and aggressive treatment of influenza with neuraminidase inhibitor antiviral drugs should be used when indicated, and data from this season show that pH1N1 remains susceptible to these agents. Currently circulating influenza A virus strains have shown resistance to amantadine and rimantadine, also known as adamantanes; therefore, adamantanes are not recommended for antiviral treatment or chemoprophylaxis of currently circulating influenza A virus strains (9).

Antiviral treatment is recommended as early as possible (ideally within 48 hours of illness onset) for patients with severe illness (e.g., patients hospitalized with influenza) or patients at high risk for serious influenza complications, including children aged <2 years, adults aged ≥65 years, and persons with certain underlying medical conditions (10).††† If treatment can be initiated within 48 hours of illness onset, antiviral medications also may be considered for outpatients with suspected or confirmed influenza who are not known to be at increased risk for developing severe illness (10).

Influenza surveillance reports for the United States are posted online weekly and are available at http://www.cdc.gov/flu/weekly. Additional information regarding influenza viruses, influenza surveillance, influenza vaccine, influenza antiviral medications, and novel influenza A infections in humans is available at http://www.cdc.gov/flu.

What is already known on this topic?

CDC collects, compiles, and analyzes data on influenza activity year-round in the United States, which show that the timing and severity of circulating influenza viruses can vary by geographic location and between influenza seasons. Although influenza causes serious illness and death every season, populations most affected at any point during a season can vary geographically, by age group, and by other characteristics.

What is added by this report?

Influenza activity in the United States began to increase in mid-November and remained elevated through February 8, 2014. During September 29, 2013–February 8, 2014, influenza A (H1N1)pdm09 viruses were identified most frequently, but a small number of influenza B and influenza A (H3N2) viruses also were reported. This season has been more severe for young and middle-aged adults than in the most recent seasons. The second highest hospitalization rates have occurred in patients aged 50–64 years this season, and more than half of influenza-associated hospitalizations occurred in adults aged 18–64 years.

What are the implications for public health practice?

Vaccination remains the most effective method to prevent influenza and its complications and is important for persons of all ages, not just the very young and old. Health-care providers should recommend and offer vaccine to all unvaccinated persons aged ≥6 months now and throughout the influenza season. Treatment with neuraminidase inhibitor antiviral medications can reduce severe outcomes of influenza, especially when initiated early, in patients with confirmed or suspected influenza.

## Footnotes

* The CDC influenza surveillance system collects five categories of information from eight data sources: 1) viral surveillance (World Health Organization collaborating laboratories, the National Respiratory and Enteric Virus Surveillance System, and novel influenza A virus case reporting); 2) outpatient illness surveillance (U.S. Outpatient Influenza-like Illness Surveillance Network); 3) mortality (122 Cities Mortality Reporting System and influenza-associated pediatric mortality reports); 4) hospitalizations (Influenza Hospitalization Surveillance Network [FluSurv-NET], which includes the Emerging Infections Program and surveillance in three additional states); and 5) summary of the geographic spread of influenza (state and territorial epidemiologist reports).

† Data reported as of February 14, 2014.

§ *Region 1:* Connecticut, Maine, Massachusetts, New Hampshire, Rhode Island, and Vermont. *Region 2:* New Jersey, New York, Puerto Rico, and the U.S. Virgin Islands. *Region 3:* Delaware, the District of Columbia, Maryland, Pennsylvania, Virginia, and West Virginia. *Region 4:* Alabama, Florida, Georgia, Kentucky, Mississippi, North Carolina, South Carolina, and Tennessee. *Region 5:* Illinois, Indiana, Michigan, Minnesota, Ohio, and Wisconsin. *Region 6:* Arkansas, Louisiana, New Mexico, Oklahoma, and Texas. *Region 7:* Iowa, Kansas, Missouri, and Nebraska. *Region 8:* Colorado, Montana, North Dakota, South Dakota, Utah, and Wyoming. *Region 9:* Arizona, California, Hawaii, Nevada, American Samoa, Commonwealth of the Northern Mariana Islands, Federated States of Micronesia, Guam, Marshall Islands, and Republic of Palau. *Region 10:* Alaska, Idaho, Oregon, and Washington.

¶ Defined as a fever (≥100°F [≥37.8°C]), oral or equivalent, and cough and/or sore throat, without a known cause other than influenza.

** The national and regional baselines are the mean percentage of visits for ILI during weeks with little or no influenza virus circulation (noninfluenza weeks) for the previous three seasons plus two standard deviations. A noninfluenza week is defined as periods of ≥2 consecutive weeks in which each week accounted for <2% of the season’s total number of specimens that tested positive for influenza. National and regional percentages of patient visits for ILI are weighted on the basis of state population. Use of the national baseline for regional data is not appropriate.

†† Activity levels are based on the percentage of outpatient visits in a jurisdiction attributed to ILI and are compared with the average percentage of ILI visits that occur during weeks with little or no influenza virus circulation. Activity levels range from minimal, which would correspond to ILI activity from outpatient clinics being at or below the average, to high, which would correspond to ILI activity from outpatient clinics being much higher than the average. Because the clinical definition of ILI is very nonspecific, not all ILI is caused by influenza; however, when combined with laboratory data, the information on ILI activity provides a clearer picture of influenza activity in the United States.

§§ Levels of activity are 1) no activity; 2) sporadic: isolated laboratory-confirmed influenza case(s) or a laboratory-confirmed outbreak in one institution, with no increase in ILI activity; 3) local: increased ILI, or at least two institutional outbreaks (ILI or laboratory-confirmed influenza) in one region of the state, with recent laboratory evidence of influenza in that region and virus activity no greater than sporadic in other regions; 4) regional: increased ILI activity or institutional outbreaks (ILI or laboratory-confirmed influenza) in at least two but less than half of the regions in the state with recent laboratory evidence of influenza in those regions; and 5) widespread: increased ILI activity or institutional outbreaks (ILI or laboratory-confirmed influenza) in at least half the regions in the state, with recent laboratory evidence of influenza in the state.

¶¶ FluSurv-NET conducts population-based surveillance for laboratory-confirmed influenza-associated hospitalizations among children aged <18 years (since the 2003–04 influenza season) and adults aged ≥18 years (since the 2005–06 influenza season). FluSurv-NET covers approximately 70 counties in the 10 Emerging Infections Program states (California, Colorado, Connecticut, Georgia, Maryland, Minnesota, New Mexico, New York, Oregon, and Tennessee) and additional Influenza Hospitalization Surveillance Project (IHSP) states. IHSP began during the 2009–10 season to enhance surveillance during the 2009 H1N1 pandemic. IHSP sites included Iowa, Idaho, Michigan, Oklahoma, and South Dakota during the 2009–10 season; Idaho, Michigan, Ohio, Oklahoma, Rhode Island, and Utah during the 2010–11 season; Michigan, Ohio, Rhode Island, and Utah during the 2011–12 season; Iowa, Michigan, Ohio, Rhode Island, and Utah during the 2012–13 season; and Michigan, Ohio, and Utah during the 2013–14 season. Incidence rates are calculated using CDC’s National Center for Health Statistics population estimates for the counties included in the surveillance catchment area. Laboratory confirmation is dependent on clinician-ordered influenza testing, and testing for influenza often is underutilized because of the poor reliability of rapid influenza diagnostic test results and greater reliance on clinical diagnosis for influenza. As a consequence, the number of cases identified as part of influenza hospitalization surveillance likely is an underestimation of the actual number of persons hospitalized with influenza.

*** The seasonal baseline proportion of P&I deaths is projected using a robust regression procedure in which a periodic regression model is applied to the observed percentage of deaths from P&I that were reported by the 122 Cities Mortality Reporting System during the preceding 5 years. The epidemic threshold is set at 1.645 standard deviations above the seasonal baseline.

††† Persons at higher risk include 1) children aged <2 years; 2) adults aged ≥65 years; 3) persons with chronic pulmonary conditions (including asthma); cardiovascular disease (except hypertension alone); renal, hepatic, hematologic (including sickle cell) disease; metabolic disorders (including diabetes mellitus); or neurologic and neurodevelopmental conditions (including disorders of the brain, spinal cord, peripheral nerve, and muscle, such as cerebral palsy, epilepsy [seizure disorders], stroke, intellectual disability [mental retardation], moderate to severe developmental delay, muscular dystrophy, or spinal cord injury); 4) persons with immunosuppression, including that caused by medications or by human immunodeficiency virus infection; 5) women who are pregnant or postpartum (within 2 weeks after delivery); 6) persons aged ≤18 years who are receiving long-term aspirin therapy; 7) American Indians/Alaska Natives; 8) persons who are morbidly obese (body mass index ≥40); and 9) residents of nursing homes and other chronic-care facilities.
